# Ovarian stromal cells as a source of cancer-associated fibroblasts in human epithelial ovarian cancer: A histopathological study

**DOI:** 10.1371/journal.pone.0205494

**Published:** 2018-10-10

**Authors:** Masayoshi Fujisawa, Aye Moh-Moh-Aung, Zheng Zeng, Teizo Yoshimura, Yoji Wani, Akihiro Matsukawa

**Affiliations:** 1 Department of Pathology and Experimental Medicine, Graduate School of Medicine, Dentistry and Pharmaceutical Sciences, Okayama University, Okayama, Japan; 2 Department of Diagnostic Pathology, Japanese Red Cross Society Himeji Hospital, Himeji, Japan; University of South Alabama Mitchell Cancer Institute, UNITED STATES

## Abstract

Fibroblasts are a major component of cancer tissue and known to contribute to cancer progression. However, it remains unknown whether they are derived from local fibroblasts or of other origin. This study was designed to identify the contribution of local stromal cells to cancer stroma in human epithelial ovarian cancer. Seventy-six cases of surgically resected primary ovarian carcinoma (48 cases confined to the ovaries and 28 cases with distant metastases) and 17 cases of secondary ovarian tumor (e.g. colon cancer metastasized to the ovary) were enrolled in this study. The tissues were immunostained for forkhead box protein L2 (FOXL2), a transcription factor crucial for ovarian development and function, and markers for cancer-associated fibroblasts (CAFs) and inflammatory cells. Under normal condition, FOXL2 expression was restricted to ovarian stromal cells and some other types of cells in female genital tracts and never found in other sites of the body. FOXL2-positive cells were found in all primary and secondary tumors in the ovary, and were the dominant stromal cells in most cases. In contrast, only a few FOXL2-positive cells were found in peritoneal seeding sites of four serous carcinoma cases, and all the other tumors at extraovarian sites had no FOXL2-positive cells. FOXL2-positive cells in the ovarian lesion variably expressed CAFs markers, such as alpha-smooth muscle actin and fibroblast activating protein, as determined by double immunostaining. Background inflammation, but not histological subtype or origin of the neoplasm seemed to correlate with the proportion of FOXL2-positive cells. These results suggest that ovarian stromal cells are the main source of cancer stroma in the ovary but do not seem to move to distant sites via circulation together with tumor cells. Our results also support the hypothesis that cancer-associated fibroblasts may originate locally, which was previously demonstrated using animal models.

## Introduction

Cancer tissue is composed of tumor cells and stromal cells, including fibroblasts, vascular endothelial cells and leukocytes. Fibroblasts–often referred to as cancer- associated fibroblasts (CAFs)–are a major component of cancer stroma and particularly important for cancer invasion and metastasis because they provide a suitable microenvironment by producing a variety of soluble factors [1].

As for human epithelial ovarian cancers, the origin of tumor cells has been intensively investigated and proposed to be epithelial cells from fallopian tubes, endometriosis, or mature teratoma [2, 3]. By contrast, little is known about the source of CAFs. Several studies based on human tissue have shown that stromal cells in ovarian cancers have features of steroid hormone-producing cells [4–6]. This finding may support the idea that ovarian CAFs are from ovarian stromal cells. On the other hand, studies based on animal models stress the importance of circulating cells such as bone marrow-derived stromal stem cells as the source of CAFs in various cancers, including ovarian, stomach, breast, and pancreatic cancer [7–11].

Cancer sometimes metastasizes to other sites of the body. However, it is also unclear whether CAFs at the primary site move and settle to distant sites with tumor cells or tumor cells migrate alone and recruit new CAFs at metastatic foci. A previous study demonstrated that fibroblasts in liver metastases of colorectal cancers have liver fibroblasts phenotype [12], however, information on this issue remains quite limited.

Unlike animal models in which the movement of cells can be easily traced using a genetic marker, it’s quite hard to trace cells in human tissues. Although epithelial cells maintain their organ-specific characteristics and antigens, stromal spindle cells look similar throughout the body and organ-specific antigens are generally lacking. CAFs have some characteristic markers such as alpha-smooth muscle actin (ASMA) and fibroblast activating protein (FAP), however, neither of them are lineage-specific; thus, the presence of these markers does not indicate their origin [13–17].

The ovary has some unique characteristics that might be helpful for understanding the origin of CAFs: (1) The ovary is an anatomically isolated organ located in the peritoneal cavity only supported by thin mesovarium, allowing us to neglect the cells directly invading from neighboring organs. (2) The ovary is normally a small-sized organ but becomes extremely large when cancer develops, indicating that almost all cancer stroma in the ovary is newly formed. (3) Ovarian stromal cells have a unique organ-specific molecule forkhead box protein L2 (FOXL2).FOXL2 is a transcription factor that is crucial for ovarian development and function [18], and expressed in ovarian stromal cells and granulosa cells from early stage of development to adult [19–24]. Although some stromal cells of female genital tract [25–27], the pituitary gland [28], and fetal eyelid [19] also express FOXL2, none of them could be a source of ovarian cancer stroma. Therefore, FOXL2 may be used as a specific marker of ovarian local stromal cells and examining stromal cells in human ovarian cancers might provide important clues as to the origin of CAFs.

In the present study, we aimed to clarify how ovarian local stromal cells contribute to cancer stroma in both ovarian and extraovarian tumors. We collected surgically resected primary ovarian cancers of four major subtypes (high-grade serous carcinoma, mucinous carcinoma, endometrioid carcinoma and clear cell carcinoma), secondary ovarian tumors (e.g. colon cancer metastasized to the ovary) and their extraovarian lesions, and then analyzed the expression of FOXL2 by immunohistochemistry. We also searched the possible factors that might affect the origin of CAFs, such as inflammation and coexisting endometriosis.

## Materials and methods

### Case selection

Seventy-six cases of surgically resected primary ovarian cancers were retrieved from the pathology files of Okayama University Hospital and Japanese Red Cross Society Himeji Hospital. Patients who underwent chemotherapy before the resection were not included in this study. All the hematoxylin and eosin (H&E)-stained slides used for diagnosis were reviewed and histological subtype of the tumor was noted. The presence of ovarian endometriosis/endometrial cysts, defined as histologically detectable endometrial tissue in the ovary, was also examined. One representative tumor slide was selected for each case and the corresponding paraffin block was used for immunohistochemical studies. Tumors whose locations could not be microscopically confirmed to be within the ovary were excluded from this study even if the preoperative diagnosis was ovarian cancer. Sixteen contralateral uninvolved ovaries among these cases were used as a normal tissue control. For the cases with metastases, paraffin blocks from the metastatic foci were also selected. Further, seventeen cases of surgically treated secondary ovarian tumors were retrieved. One block from the ovary and one block form the primary organ (e.g. colon) were used for each case. Patients who underwent chemotherapy within three months of the surgery were excluded. The protocol for this study was reviewed and approved by the institutional review boards of Okayama University (1608–008) and Japanese Red Cross Society Himeji Hospital (2016–14). Although individual written consents were not obtained, we disclosed the study plan on our website, providing the patients or their families with the opportunity to opt out, and only the cases without their refusal were enrolled in the study. This consent procedure conformed to amended Ethical Guidelines for Clinical Studies provided by Ministry of Health, Labor and Welfare of Japan (May 31, 2015) and was approved by the institutional review boards. The number of cases for each condition is listed on Table 1.

**Table 1:** The summary of cases.

Histologic type	Location		
	Only the ovary	The ovary+Extraovarian site	Total
Primary ovarian epithelial cancer			
High-grade serous carcinoma	5	15	20
Mucinous carcinoma	13	5	18
Endometrioid carcinoma	19	2	21
Clear cell carcinoma	11	6	17
(Subtotal)	48	28	76
Secondary ovarian tumor			
Colorectal adenocarcinoma		11	11
Gastric adenocarcinoma		5	5
Pancreatic adenocarcinoma		1	1
(Subtotal)		17	17
Total	48	45	93

### Immunohistochemistry

Serial sections of 4μm thickness were prepared from formalin-fixed and paraffin-embedded blocks collected as described above. One section was stained with H&E and others were used for immunohistochemistry for each block. Immunostaing was performed manually by a conventional method: Briefly, sections were deparaffinized in xylene and rehydrated in a sequence of descending concentrations of ethanol. Endogenous peroxidase reactivity was blocked with 3% H_2_O_2_ for 10 minutes. For antigen retrieval, sections were enclosed in a pressure cooker with citrate buffer (pH6.0) or EDTA solution (pH8.0) and microwaved (500W) continuously for 20 minutes. Sections were then incubated with a respective primary antibody for 1.5 hour at room temperature (Table 2). After washing, sections were treated with a matching secondary antibody according to the manufacturer’s instructions. Histochemical reactions were developed using 3, 3-diaminobenzidine (DAB) or Vector Red as the chromogenic substrate for peroxidase or alkaline phosphatase, respectively. Finally sections were counterstained with hematoxylin, dehydrated, and mounted. For double immunostaining, an additional immunostaining was performed before counterstaining. The additional immunohistochemistry was started by antigen retrieval, followed by primary and secondary antibody incubation, and completed with histochemisty with a chromogen different from the former staining. Positive and negative controls were run with appropriate tissue sections. The primary antibodies and the staining conditions are detailed on Table 2 and protocols.io (http://dx.doi.org/10.17504/protocols.io.s8dehs6).

**Table 2:** Antibody and staining conditions.

Antibody	Species	Clone/Code No.	Manufacturer	Dilution	Pretreatment	Detection System	Chromogen (color)
FOXL2	goat	NB100-1277	Novus Biologicals, Littlecon, CO	1:400	Citrate pH6	Polink-2 Plus HRP^a^	DAB(brown)
ASMA	mouse	1A4	Dako, Carpinteria, CA	1:100	None	ImmPRESS-AP^b^	Vector Red (red)^b^
FAP	rabbit	NB100-58755	Novus Biologicals, Littlecon, CO	1:100	Citrate pH6	ImmPRESS-AP^b^	Vector Red (red)^b^
CD138	mouse	B-A38	Cell Marque, Rocklin, CA	1:100	EDTA pH8	Simple Stain MAX PO^c^	DAB(brown)
CD163	mouse	10D6	Novocastla, Newcastle, UK	1:200	EDTA pH8	Simple Stain MAX PO^c^^d^	DAB(brown)^d^
CD45	mouse	2B11+PD7/26	Dako, Carpinteria, CA	1:100	Citrate pH6	ImmPRESS-AP^b^	Vector Red (red)^b^
CD31	mouse	JC70A	Dako, Carpinteria, CA	1:40	EDTA pH8	ImmPRESS-AP^b^	Vector Red (red)^b^
Collagen type4	mouse	CIV22	Cell Marque, Rocklin, CA	1:500	Citrate pH6^e^	ImmPRESS-AP^b^	Vector Red (red)^b^

All the above mouse antibodies are monoclonal and the rabbit/goat antibodies are polyclonal.

^a^GBI labs, Bothell, WA.

^b^Vector laboratories, Burlingame, CA.

^c^Nichirei Biosciences, Tokyo, Japan.

^d^For the double staining with FOXL2, CD163 was visualized with ImmPRESS-AP and Vector Red (red).

^e^This microwave treatment was followed by Proteinase K treatment (0.002% Proteinase K, 3minutes, in room temperature). Proteinase K was obtained from Roche, Mannheim, Germany (Cat.No.03 115 887 001).

### Histological evaluation

FOXL2 expression was scored using a 5-tiered scale based on the percentage of immunoreactive stromal cells in the examined area: 0, 0% positive; 1+, less than 9% positive; 2+, 10% to 33% positive; 3+, 34% to 66% positive, 4+, more than 67% positive. Based on previous reports from others [20–23], only nuclear staining was regarded as positive irrespective of staining intensity. All the stromal cells including fibroblasts, endothelial cells and inflammatory cells within 0.5mm from the outermost tumor cells were accounted but necrotic areas and extremely inflamed areas were ignored. All the ovarian and extraovarian lesions were enrolled in this analysis.

CD31, collagen type4, CD45 and CD163 were double-stained with FOXL2 to determine whether endothelial cells, vascular smooth muscle cells, and inflammatory cells expressed FOXL2. A total of 12 samples (two samples from each category: normal, serous, mucinous, endometrioid, clear, secondary) were stained and analyzed microscopically.

The expression of ASMA and FAP by FOXL2-positive cells was also determined by double staining. The expression level was scored using a 5-tiered scale that was based on the percentage of immunoreactive cells among FOXL2-positive stromal cells: 0, 0% positive; 1+, less than 9% positive; 2+, 10% to 33% positive; 3+, 34% to 66% positive, 4+, more than 67% positive. For these markers, definitive cytoplasmic staining was regarded positive. All the ovarian lesions were enrolled in this analysis.

Two pathologists (M.F. and A.M.) scored the above semi-quantitative analyses independently. For the cases with discrepancy, the slides were reviewed using a multiheaded microscope to achieve a consensus.

Plasma cells and macrophages were highlighted by the expression of CD138 and CD163 respectively. The number of these cells in one high power field (objective lens: x40 UPlanAPO, Olympus) was counted where these cells had aggregated the most densely in a given area. Each tumor tissue was divided into the adjacent area (within 0.5mm from outmost tumor cells) and the distant area (stroma apart from tumor cells, such as capsule), and evaluated separately. Slides without the tumor cell-free distant area (four cases of Krukenberg tumor where tumor cells infiltrate diffusely throughout the ovary) were excluded and all the other ovarian lesions were enrolled in this analysis.

### Statistical analysis

Statistical analysis was performed with Prism 7 (GraphPad Software, San Diego, CA). We used Mann-Whitney test for comparing immunostaining scores between ovarian and extraovarian lesions and Kruskal-Wallis test for comparing among histological subtypes. The presence of endometriosis/endometrial cysts and inflammation are compared with proportion of FOXL2-positive cells with Fisher’s exact probability test and Mann-Whitney test, respectively. Values for *p* < 0.05 were considered to be statistically significant.

## Results

### FOXL2-positive stromal cells are abundant in the stroma of ovarian lesions

**Fig 1:**
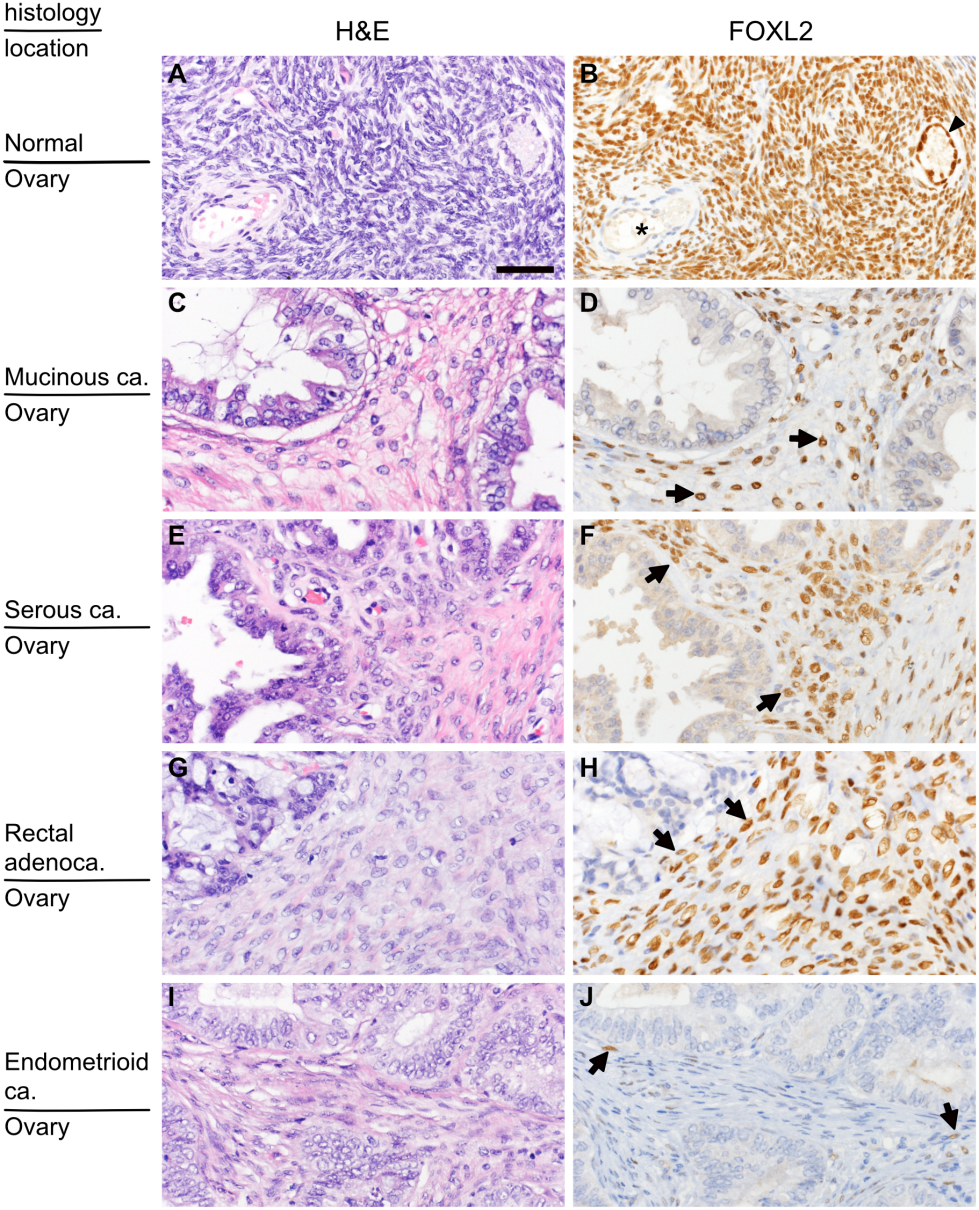
Distribution of FOXL2-positive stromal cells in the ovary. (A and B) Normal ovarian cortex. Almost all stromal cells as well as granulosa cells (arrow head) show nuclear positivity for FOXL2. By contrast, none of the cells that constitute blood vessels (asterisk) have nuclear FOXL2. (C and D) Primary mucinous carcinoma. (E and F) Primary serous carcinoma. (G and H) Secondary ovarian tumor (ovarian metastasis of rectal adenocarcinoma). Most stromal spindle cells show nuclear positivity for FOXL2. (I and J) Primary endometrioid carcinoma, a rare example of primary ovarian cancer containing only a few FOXL2-postive cells. H&E (left panels) and FOLX2 immunostaining of the corresponding area (right panels; only nuclear staining is considered positive). Arrows indicate FOXL2-positive cells. The bar in (A) indicates 50μm, and the magnification is identical for all the pictures.

In normal ovaries, almost all ovarian stromal cells as well as granulosa cells expressed FOXL2 (Fig 1A and 1B). By contrast, other cells such as endothelial cells, vascular smooth muscle cells and inflammatory cells were negative for FOXL2, as confirmed by double immunostaining (S1 Fig). Similar to normal ovary, in primary and secondary ovarian cancers, FOXL2 was expressed by fibroblast-like spindle cells, but not by endothelial cells, vascular smooth muscles or inflammatory cells (S1 Fig). The majority of stromal cells were FOXL2-positive in most cases (Fig 1C–1H). In a few cases of endometrioid carcinoma (5 out of 21) and serous carcinoma (2 out 21), a small number of FOXL2-positive stromal cells were detected (Fig 1I and 1J). There was no case without FOXL2-positive stromal cells in the ovarian lesion. In contrast to stromal cells, tumor cells did not express FOXL2 except for five cases (5.4%) in which some tumor cells had FOXL2 staining, which was thought to represent aberrant expression.

### FOXL2-positive cells are mostly absent in the stroma of extraovarian lesions

**Fig 2:**
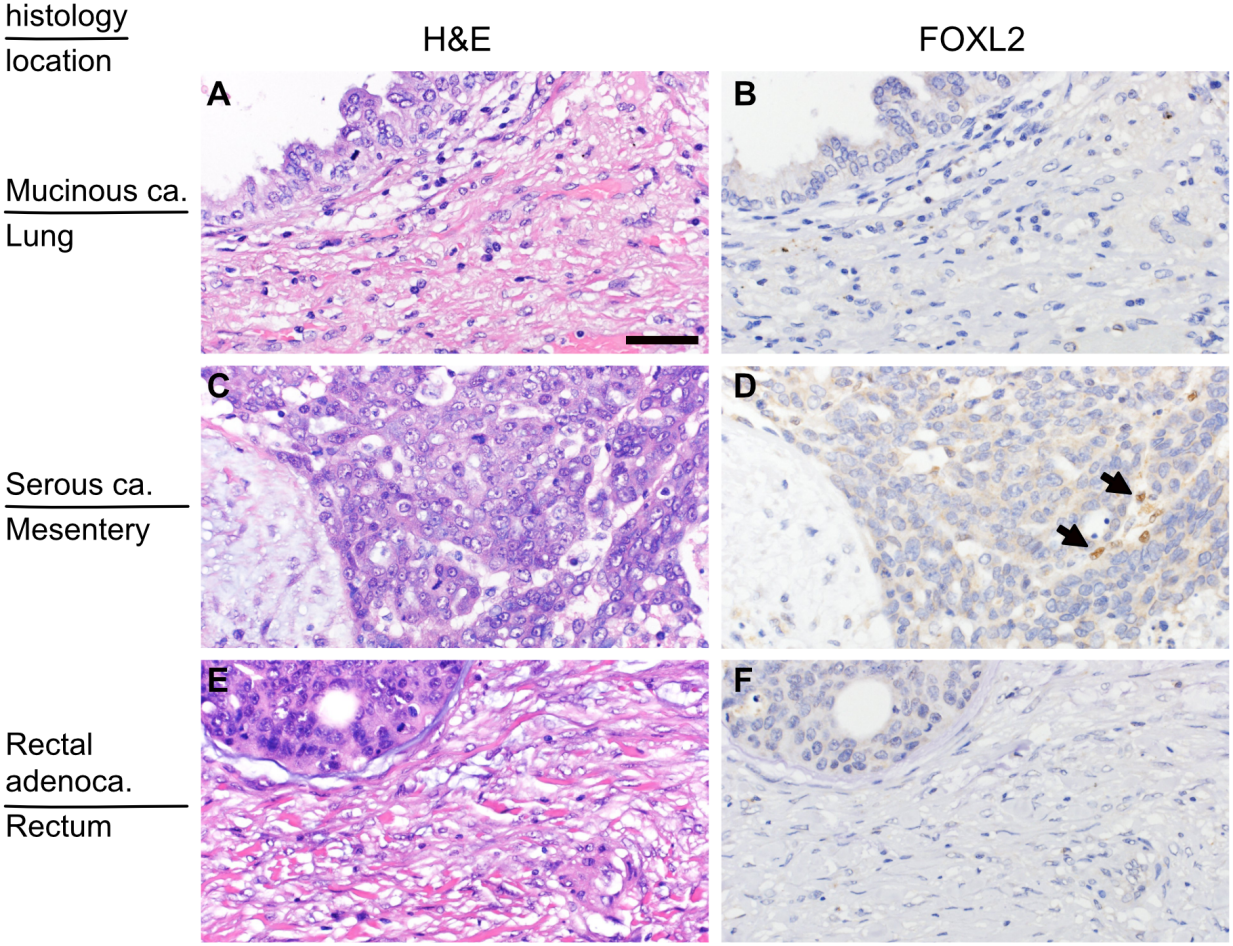
Distribution of FOXL2-positive stromal cells in extraovarian lesions. (A and B) A metastatic site of mucinous ovarian carcinoma in the lung (the same case as Fig 1C and 1D). There are no FOXL2-positive stromal cells. (C and D) A peritoneal seeding site of serous ovarian carcinoma (the same case as Fig 1E and 1F). There are few FOXL2-positive cells. (E and F) The primary site of rectal cancer that caused secondary ovarian tumor (the same case with Fig 1G and 1H). There are no FOXL2-positive stromal cells. H&E (left panels) and FOLX2 immunostaining of the corresponding area (right panels). Arrows indicate FOXL2-positive cells. The bar in (A) indicates 50μm, and the magnification is identical for all the pictures.

We next examined the expression of FOXL2 in the metastatic foci of the primary ovarian cancers in the lung, brain, lymph node and peritoneum, including omentum, mesentery and uterine surface, and the primary lesions of the secondary ovarian tumors of colon, rectum, stomach and pancreas origin. Among these extraovarian tissues, most of endometrial/endosalpingeal stromal cells and some smooth muscle cells of the uterus and the fallopian tube expressed FOXL2, but the remaining tissues completely lacked FOXL2-positive cells under normal condition (S2 and S3 Figs). Similarly, there were no FOXL2-positive cells in most of extraovarian lesions (metastatic foci) of the primary ovarian cancers (Fig 2A and 2B). However, in a few cases of peritoneal metastases of serous carcinoma (4 out of 15), a small portion (less than 5%) of fibroblast-like spindle cells showed FOXL2 positivity (Fig 2C and 2D). In one of these four cases, very few FOXL2-positive cells were present close to psammoma bodies (a trace of past tumor tissue) but not around viable tumor cells (S4 Fig). All the primary foci of secondary ovarian tumor didn’t contain FOXL2-positive cells (Fig 2E and 2F).

### The proportions of FOXL2-positive cells are similar among histological subtypes

**Fig 3:**
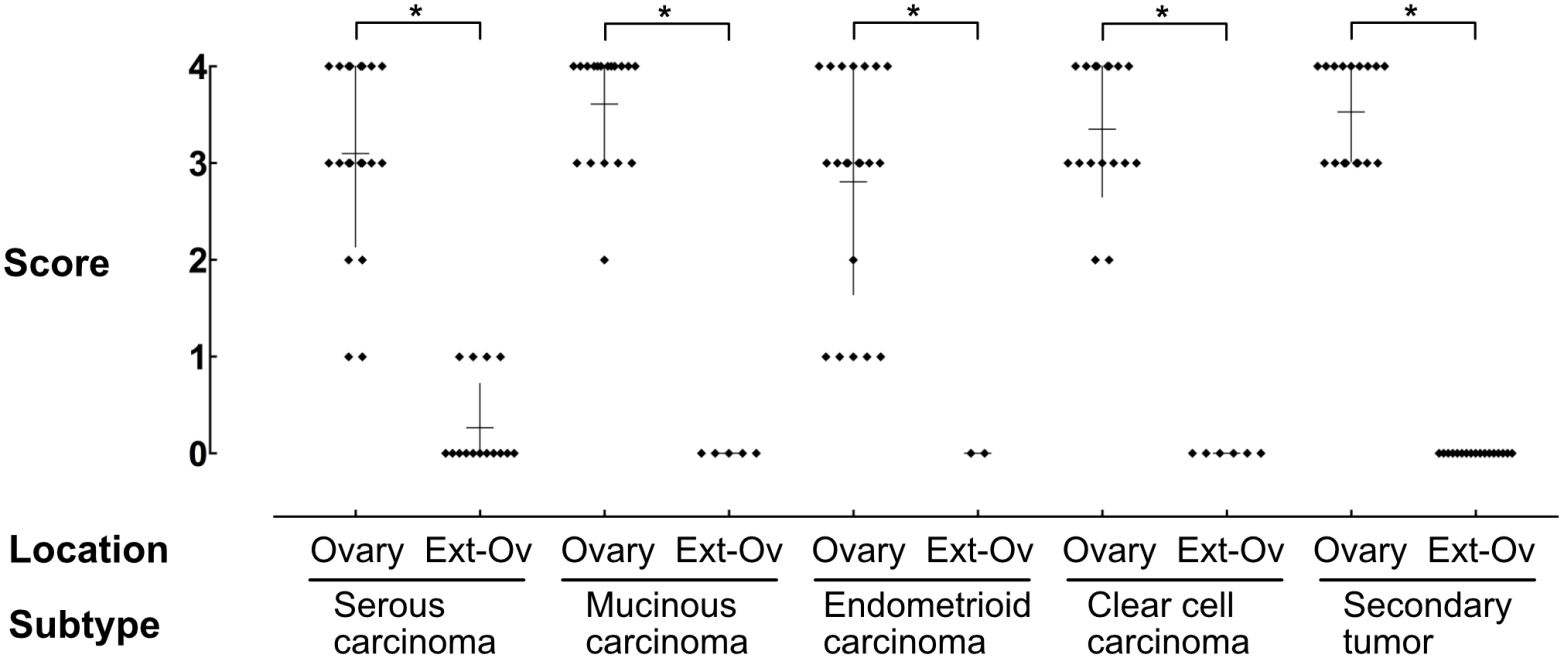
The proportion of FOXL2-positive cells in cancer stroma. The proportion of FOXL2-positive cells on each tissue section was scored using 5-tired scale and the results were plotted. High percentages of cancer stromal cells were FOXL2-positive in the ovary, whereas there were almost no FOXL2-positive cells outside the ovary (Ext-O). Asterisks indicate a significant difference between the groups by Mann-Whitney test (*p* < 0.05).

Since the proportion of FOXL2-positive cells in cancer stroma varied among cases, we then examined semi-quantitatively whether it depends on histological subtypes or tumor locations. The percentage of FOXL-positive cells in cancer stroma was scored from 0 (no positive cells) to 4 (more than 67% positive) and compared statistically. Although a few endometrioid and serous carcinoma cases had a small number of FOXL2-positive cells, the proportion of FOXL2-positive cells in the ovary did not differ among histological subtypes (Kruskal-Wallis test). By contrast, the difference between ovarian and extraovarian lesions was statistically significant (Mann-Whitney test) for all histological subtypes (Fig 3).

### ASMA and FAP are expressed by FOXL2-positive ovarian cancer stromal cells

**Fig 4:**
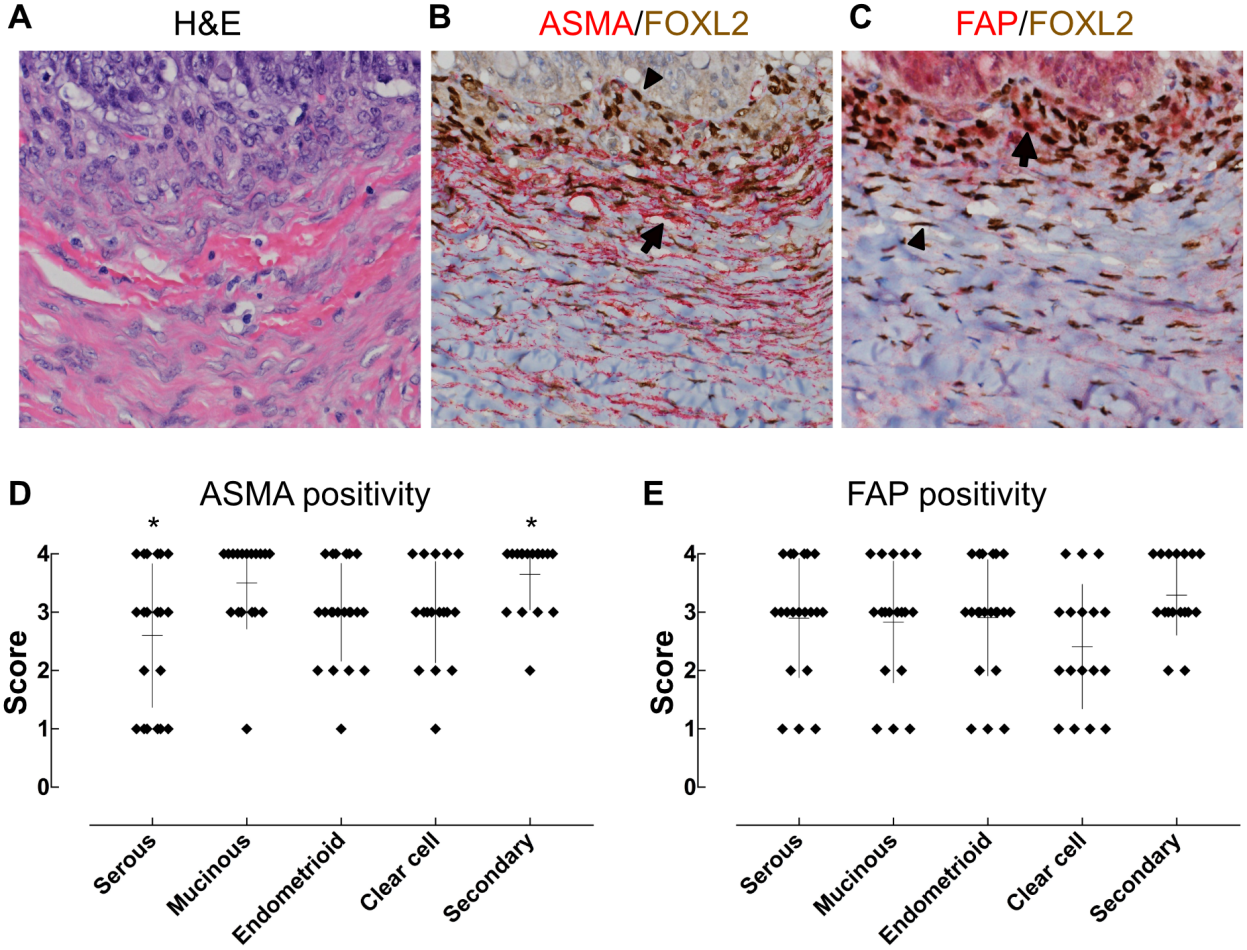
Expression of ASMA and FAP by FOXL2-positive ovarian cells. (A-C) Tumor tissues were double stained for FOXL2 and ASMA or FAP. Nuclear FOXL2 was stained brown whereas cytoplasmic ASMA and FAP were stained red in B and C, respectively. Some FOXL2-positive cells were also stained positive for ASMA or FAP (arrows), but others were negative (arrow heads). (D and E) The frequency of ASMA and FAP positive cells in FOXL2-positive cells. ASMA and FAP were variously but at least focally expressed by FOXL2-positive cells of all cases. Asterisks indicate a significant difference between two groups (Kruskal-Wallis test followed by multiple comparison).

As described above, CAFs are often stained positively for ASMA and FAP. To evaluate whether FOXL2-positive cells in ovarian cancer stroma express these CAF markers, we performed double immunostaining on all the primary and secondary ovarian lesions. ASMA and FAP were variously but at least focally expressed in FOXL2-positive cells in all cases (Figs 4A–4C). The proportion of such cells did not differ greatly among histological subtypes, although a difference of ASMA expression between serous carcinomas and secondary tumors was noted statistically (Fig 4D and 4E). Thus, FOXL2-positive cells in ovarian cancers seemed to have the capacity to express CAF markers irrespective of cancer subtypes.

### The number and location of plasma cells correlated with the proportion of FOXL2-positive cells in ovarian cancer stroma

**Fig 5:**
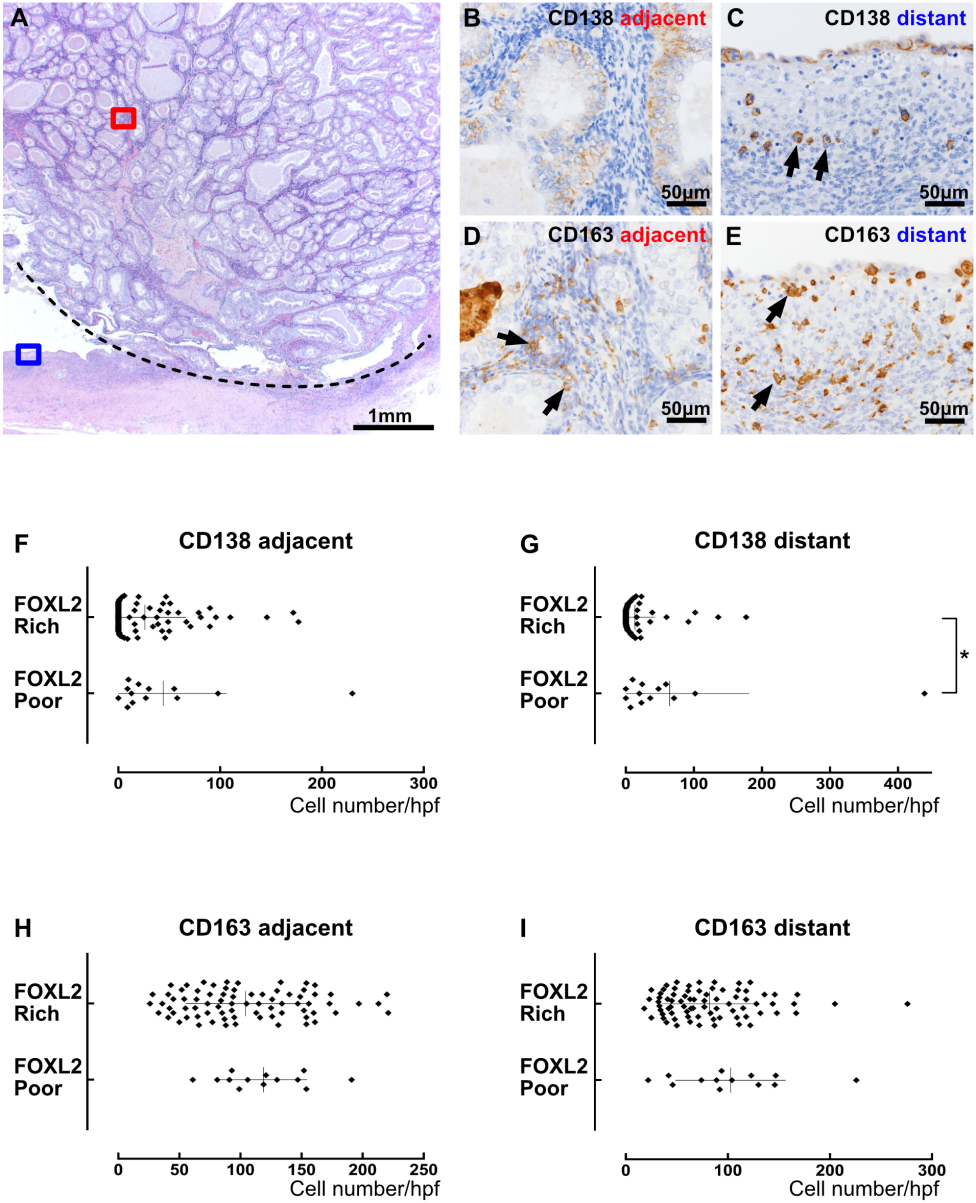
Correlation between chronic inflammation and FOXL2-positive cells in ovarian cancer stroma. (A-E) An example of endometrioid carcinoma where FOXL2-positive stromal cells were scant (FOXL2-poor). (A) Low magnification of H&E. The area above the dotted line is cancer tissue and the area below the line is a pre-existing endometrial cyst. (B and C) CD138 immunostaining. Tumor area (B: red square in A) contained no plasma cells and nontumor area (C: blue square in A) had a few plasma cells (arrows). (D and E) CD163 immunostaining. Both tumor area (D) and nontumor area (E) had some macrophages (arrows). (F-I) The number of CD138-positive plasma cells (F and G) or CD163-positive macrophages (H and I) in one high power field was plotted, comparing between cases with FOXL2-poor stroma (score1 and 2) and those with FOXL2-rich stroma (score 3 and 4). The presence of plasma cells apart from tumor cells correlated with FOXL2-positive cell population, whereas the presence of plasma cells near tumor cells or macrophages in any place did not. The asterisk indicates a significant difference between the groups by Mann-Whitney test (*p* < 0.05).

To determine the possible contribution of endometriosis or chronic inflammation to the expression of FOXL2 by ovarian cancer stromal cells, we compared the presence of endometriosis and the number of CD138-positive plasma cells and CD163-positive macrophages between FOXL2-poor (score1, 2) and FOXL2-rich (score 3, 4) cases. The presence of endometriosis was histologically evident in 32 cases of 93 ovarian cancers (34%), but there was no correlation between the presence of endometriosis and the FOXL2-positivity scores (S1 Table). The number of plasma cell and macrophages infiltrating in the area adjacent to or distant from tumor cells were counted separately, because the numbers in the two areas were found greatly different in some cases. FOXL2-poor cases (n = 13) contained more plasma cells than FOXL2-rich cases (n = 76) in the distant areas. By contrast, the number of plasma cells did not differ between FOXL2-poor and FOXL2-rich cases in the adjacent areas (Fig 5). The number of macrophages did not differ between FOXL2-poor cases and FOXL2-rich cases in either area. Thus, only the presence of plasma cells distant from tumor cells seemed to correlate with decreased proportion of FOXL2-positive cells in the ovarian cancer stroma.

## Discussion

In the present study, we histologically analyzed the presence of local stromal cells in human ovarian cancer tissues by examining the expression of FOXL2, a marker virtually specific to ovarian stromal cells. The majority of primary and secondary ovarian cancers had abundant FOXL2-positive cells in ovarian lesions whereas there were no FOXL2-positive cells in extraovarian lesions except for four peritoneal seeding cases. Many of FOXL2-positive cells in ovarian lesions also expressed ASMA and FAP, showing they have a CAF phenotype. The presence of plasma cells distant from neoplasm, but not the histological subtype correlated with the proportion of FOXL2-positive cells.

The expression of FOXL2 is restricted to the ovary and other female genital organs under normal condition. In fact, we found no FOXL2 positive cells in other organs examined. Ovarian granulosa cells also express FOXL2, but they can be excluded as a source of CAFs because they are structurally separated from cancer cells by follicular basement membranes and the number of granulosa cells decrease dramatically with age by follicular atresia [29, 30]. As for the uterus and the fallopian tubes, FOXL2 expression was limited to the luminal side (i.e. endometrium, tubal mucosa, and inner part of the myometrium/myosalpinx), not affecting the analysis of the metastatic foci located on serosal surface.

Our finding that FOXL2-positive cells are predominant in the stroma of ovarian tumors is consistent with the results of previous studies in which hormonal activities of ovarian cancer stroma were examined. Hormonal activities of stromal cells may suggest their ovarian origin; however, hormonal activities of cancer stroma differed from those of ovarian stroma distant form cancer [5]. Therefore, FOXL2 expression seems to be more stable than hormonal activities. In any case, our results focusing on the expression of FOXL2 support the idea that ovarian stromal cells are the origin of ovarian cancer stromal cells.

Bone marrow-derived stromal stem cells are thought to be the source of CAFs and there is a possibility that such stem cells or other circulating cells begin to express FOXL2 when they settle in the ovary. If tumor cells attract circulating stem cells and induce them to become CAFs, the phenotype of CAFs is expected to be similar irrespective of the location. However, the expression of FOXL2 differed greatly between ovarian and extraovarian lesions. Therefore, circulating stem cells are highly unlikely to be the origin of FOXL2-positive CAFs in ovarian cancers.

Little is known whether CAFs in the primary cancer tissue move to remote organs together with metastasizing tumor cells. Although primary ovarian cancers contained abundant FOXL2-positive cells, there were no FOXL2-poitive cells in most metastatic sites. We found only four exceptional cases of serous carcinoma that contained a small number of FOXL2-positive cells at peritoneal seeding sites. These results might suggest that local fibroblasts could only occasionally “metastasize” with cancer cells via direct seeding of the body cavity whereas they will never metastasize via lymphatic spread and hematogenous spread among three metastasis pathways [31]. Conversely, when colon or other extraovarian-primary cancer without FOXL2-positive fibroblasts metastasized to the ovary, there were abundant FOXL2-positive fibroblasts in the secondary ovarian tumors. Other researchers had also pointed out that some stromal cells of ovarian metastases from colorectal cancer have alpha-inhibin, another ovarian stroma marker, not as specific or sensitive as FOXL2 [32], and SF-1 [5]. These facts might indicate that CAFs in colorectal cancer do not traffic with tumor cells and that tumor cells induce the proliferation of local stromal cells at the metastatic foci. Our results are consistent with the previous report showing the origin of fibroblasts in liver metastases of colorectal cancer [12]. However, there remains a possibility that CAFs actually “metastasize” to distant organs but change their FOXL2 status there: i.e. ovarian CAFs lose FOXL2 expression at extraovarian sites and colorectal CAFs gain FOXL2 expression in the ovary. Although this may be unlikely based on the physiological character of FOXL2 and the clear-cut difference of FOXL2 expression by location, further studies including animal experiments are required to exclude this possibility.

A few cases of ovarian lesions did not have many FOXL2-positive cells and FOXL2-negative fibroblasts predominated in those lesions. The origin of such FOXL2-negative fibroblasts remains unknown. We showed that FOXL2-poor cases had a significantly larger number of plasma cells in the areas distant from cancer cells such as tumor capsules or neighboring cyst walls, when compared with FOXL2-rich cases. Since the infiltration of plasma cells is an indicator of chronic inflammation [33–35], and endometriosis is frequently accompanied with chronic inflammation [36], the presence of chronic inflammation in the background ovary might account for the presence of FOXL2-negative fibroblasts. However, it seems far from the answer and further studies including animal models are necessary to determine the origin of FOXL2-negative fibroblasts.

Although ovarian stromal cells have morphological features of fibroblasts [30], some investigators stressed that they are different from fibroblasts of other organs [37]. We speculate that ovarian stromal cells are a kind of fibroblasts specialized for folliculogenesis and supporting cyst formation as well as hormone production. For example, primary ovarian carcinomas tend to be cystic and their metastatic lesions are not usually cystic. Conversely, colorectal adenocarcinomas are seldom cystic at their primary sites but often become cystic when they metastasize to the ovary [32]. Additional studies are necessary to determine whether our findings are limited to ovarian cancers or applicable to cancers of other organs. As for animal models, one study recently showed that local fibroblasts are the major source of CAFs [38], although this idea is still in a minority group.

In conclusion, our results strongly suggest that local stromal cells are the major source of fibroblasts in ovarian cancers and they do not seem to traffic with tumor cells via circulation although they occasionally accompany with peritoneal seeding. We also found a negative correlation between the chronic inflammation of the background ovary and the proportion of FOXL2-positive fibroblasts in the cancer stroma. Although our results obtained with a series of human cancer tissues does not explain the origin of all CAFs and might be limited to the ovary, they support the hypothesis that CAFs originate from local fibroblasts, which have been shown in a few animal models.

## Supporting information

S1 Fig Double staining of FOXL2 and stromal cell markers. (A-D) Normal ovary, (E-H) Mucinous carcinoma, (I-L) Serous carcinoma, (M-P) Secondary tumor, (Q-T) Endometrioid carcinoma (the same cases as Fig 1). CD31 immunostaining (A,E,I,M,Q) highlights endothelial cells red (arrows), and the nuclei of these cells are not stained with FOXL2 (brown). Collagen type4 (B,F,J,N,R) is a major component of basement membrane. Vascular smooth muscle cells are completely embedded in dense basement membranes (arrows), whereas fibroblasts are only partially and loosely surrounded by thin basement membranes (arrow heads). The nuclei of vascular smooth muscle cells are not stained with FOXL2. CD45 is a pan-leukocyte marker (C,G,K,O,S) and CD163 is specific to differentiated macrophages (D,H,L,P,T). The nuclei of inflammatory cells labeled with these markers (arrows) are not stained with FOXL2. Only nuclear brown staining is considered positive for FOXL2. Note some endothelial, smooth muscle, and inflammatory cells have condensed nuclei but not stained brown. The bar in (A) indicates 50μm, and the magnification is identical for all the pictures.
(TIFF)

S2 Fig Distribution of FOXL2-positive stromal cells in female genital tract under normal condition. (A and B) Luminal side of the uterus. Most endometrial stromal cells (arrow) and adjacent myometrial smooth muscle cells (arrow head) are positive for FOXL2. (C and D) Serosal side of the uterus. Smooth muscle cells in the deep myometrium don’t express FOXL2. (E and F) Luminal side of the fallopian tube. Most of the mucosal stromal cells express FOXL2 (arrow). (G and H) Serosal side of the fallopian tube. Mesothelial cells (arrow) and smooth muscle cells beneath the serosal surface (arrowhead) don’t show FOXL2 positivity. H&E (left panels) and FOLX2 immunostaining of the corresponding area (right panels; only nuclear staining is considered positive). The bar in (A) indicates 50μm, and the magnification is identical for all the pictures.
(TIFF)

S3 Fig Expression of FOXL2 in organs other than female genital tract under normal condition. Besides female genital tract, no cells showed nuclear FOXL2 expression in the organs examined in this study. H&E (left panels) and FOLX2 immunostaining of the corresponding area (right panels; only nuclear staining is considered positive). Locations are indicated on the far left. The bar indicates 50μm, and the magnification is identical for all the pictures.
(TIFF)

S4 Fig A case of serous carcinoma with few FOXL2-positive cells in a peritoneal seeding site. Very few FOXL2-positive cells (arrows) are present near psammoma bodies (arrowheads) whereas most of the stromal cells in this metastatic lesion did not show FOXL2 positivity including those around viable tumor cells (asterisks). The bar indicates 50μm, and the magnification is identical for all the pictures.
(TIFF)

S1 Table FOXL2 proportion in cancer stoma and the presence of endometriosis. The presence of endometriosis was compared between FOXL2-poor (score1, 2) and FOXL2-rich (score3, 4) cases. FOXL2 proportion in cancer stroma did not seem to correlate with the presence of ipsilateral endometriosis or endometriosis in any part.
(XLSX)
