# Supplementary figures and images for: Ovarian stromal cells as a source of cancer-associated fibroblasts in human epithelial ovarian cancer: A histopathological study
Source: PLoS One. 2018 Oct 10;13(10):e0205494. doi: 10.1371/journal.pone.0205494 (PMC6179287; doi:10.1371/journal.pone.0205494)

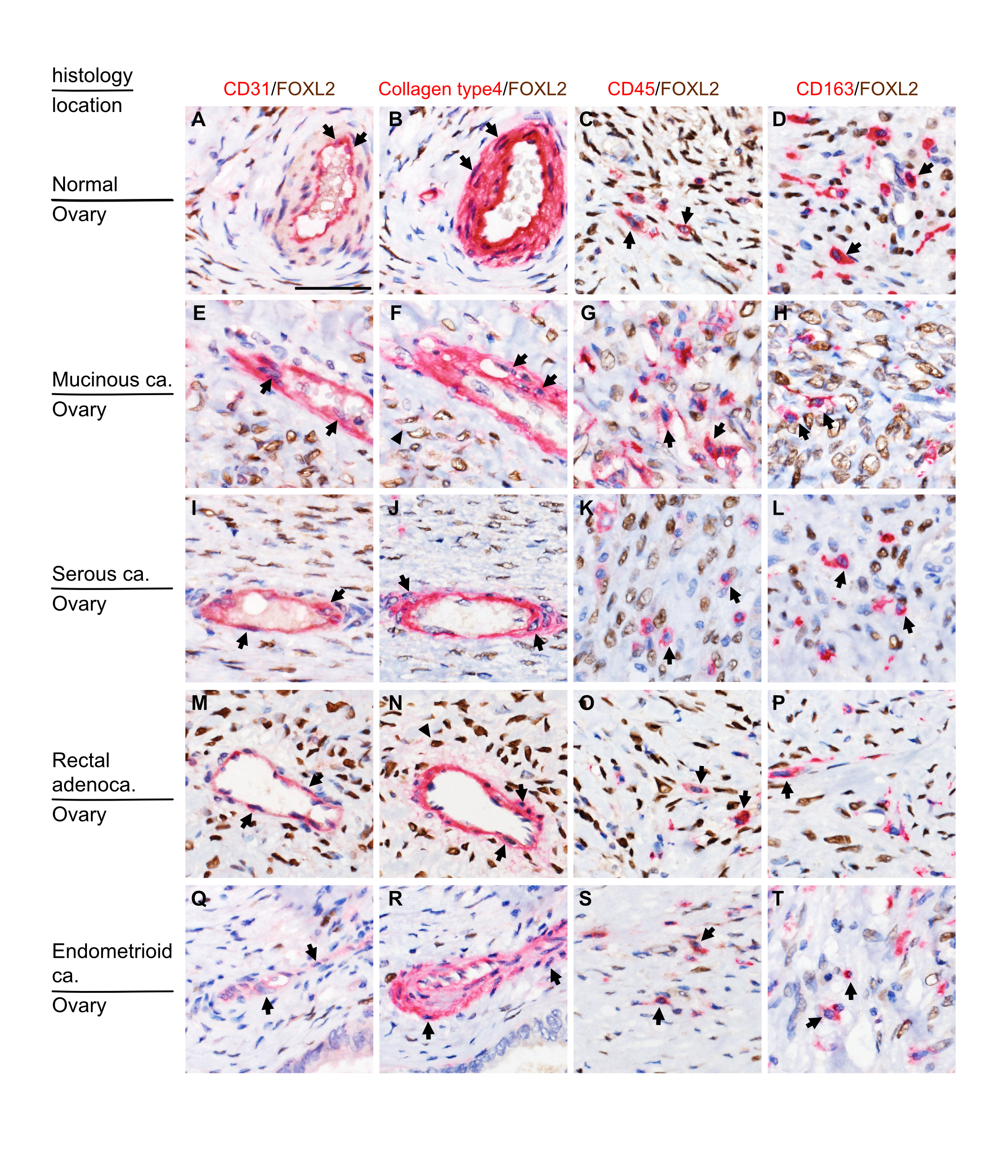

Supplement: S1 Fig — (A-D) Normal ovary, (E-H) Mucinous carcinoma, (I-L) Serous carcinoma, (M-P) Secondary tumor, (Q-T) Endometrioid carcinoma (the same cases as Fig 1). CD31 immunostaining (A,E,I,M,Q) highlights endothelial cells red (arrows), and the nuclei of these cells are not stained with FOXL2 (brown). Collagen type4 (B,F,J,N,R) is a major component of basement membrane. Vascular smooth muscle cells are completely embedded in dense basement membranes (arrows), whereas fibroblasts are only partially and loosely surrounded by thin basement membranes (arrow heads). The nuclei of vascular smooth muscle cells are not stained with FOXL2. CD45 is a pan-leukocyte marker (C,G,K,O,S) and CD163 is specific to differentiated macrophages (D,H,L,P,T). The nuclei of inflammatory cells labeled with these markers (arrows) are not stained with FOXL2. Only nuclear brown staining is considered positive for FOXL2. Note some endothelial, smooth muscle, and inflammatory cells have condensed nuclei but not stained brown. The bar in (A) indicates 50μm, and the magnification is identical for all the pictures. (TIFF) [file pone.0205494.s001.tiff]

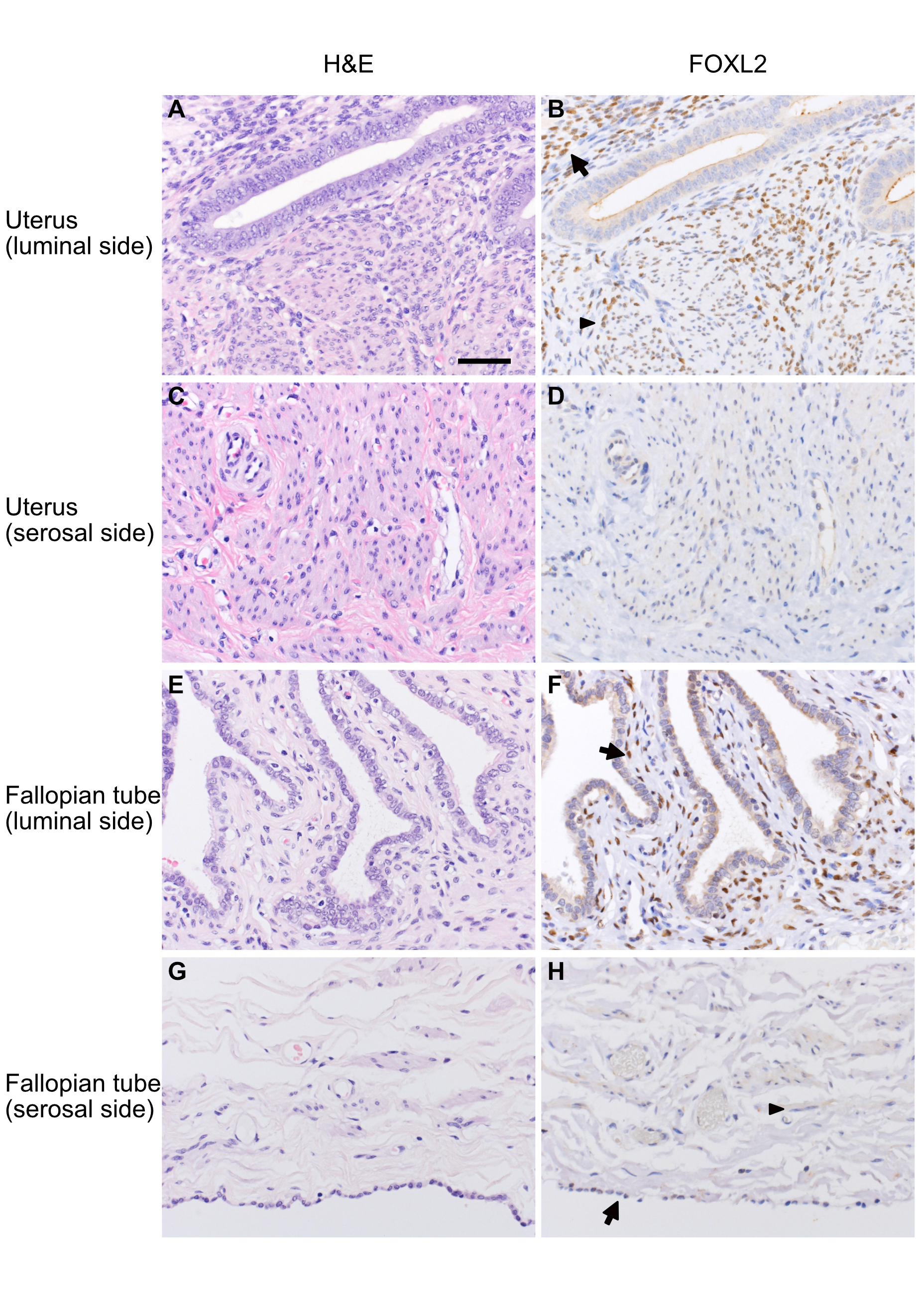

Supplement: S2 Fig — (A and B) Luminal side of the uterus. Most endometrial stromal cells (arrow) and adjacent myometrial smooth muscle cells (arrow head) are positive for FOXL2. (C and D) Serosal side of the uterus. Smooth muscle cells in the deep myometrium don’t express FOXL2. (E and F) Luminal side of the fallopian tube. Most of the mucosal stromal cells express FOXL2 (arrow). (G and H) Serosal side of the fallopian tube. Mesothelial cells (arrow) and smooth muscle cells beneath the serosal surface (arrowhead) don’t show FOXL2 positivity. H&E (left panels) and FOLX2 immunostaining of the corresponding area (right panels; only nuclear staining is considered positive). The bar in (A) indicates 50μm, and the magnification is identical for all the pictures. (TIFF) [file pone.0205494.s002.tiff]

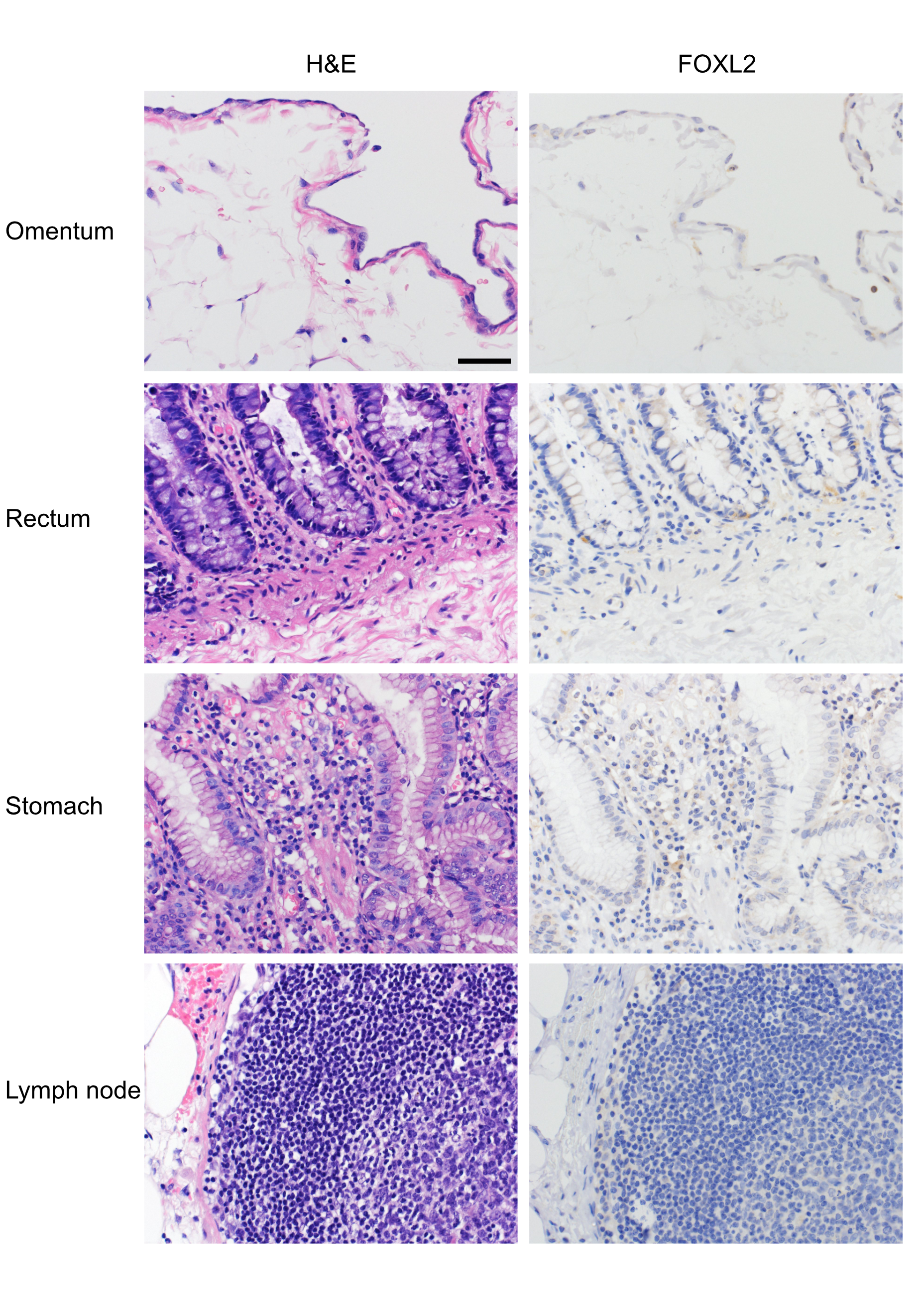

Supplement: S3 Fig — Besides female genital tract, no cells showed nuclear FOXL2 expression in the organs examined in this study. H&E (left panels) and FOLX2 immunostaining of the corresponding area (right panels; only nuclear staining is considered positive). Locations are indicated on the far left. The bar indicates 50μm, and the magnification is identical for all the pictures. (TIFF) [file pone.0205494.s003.tiff]

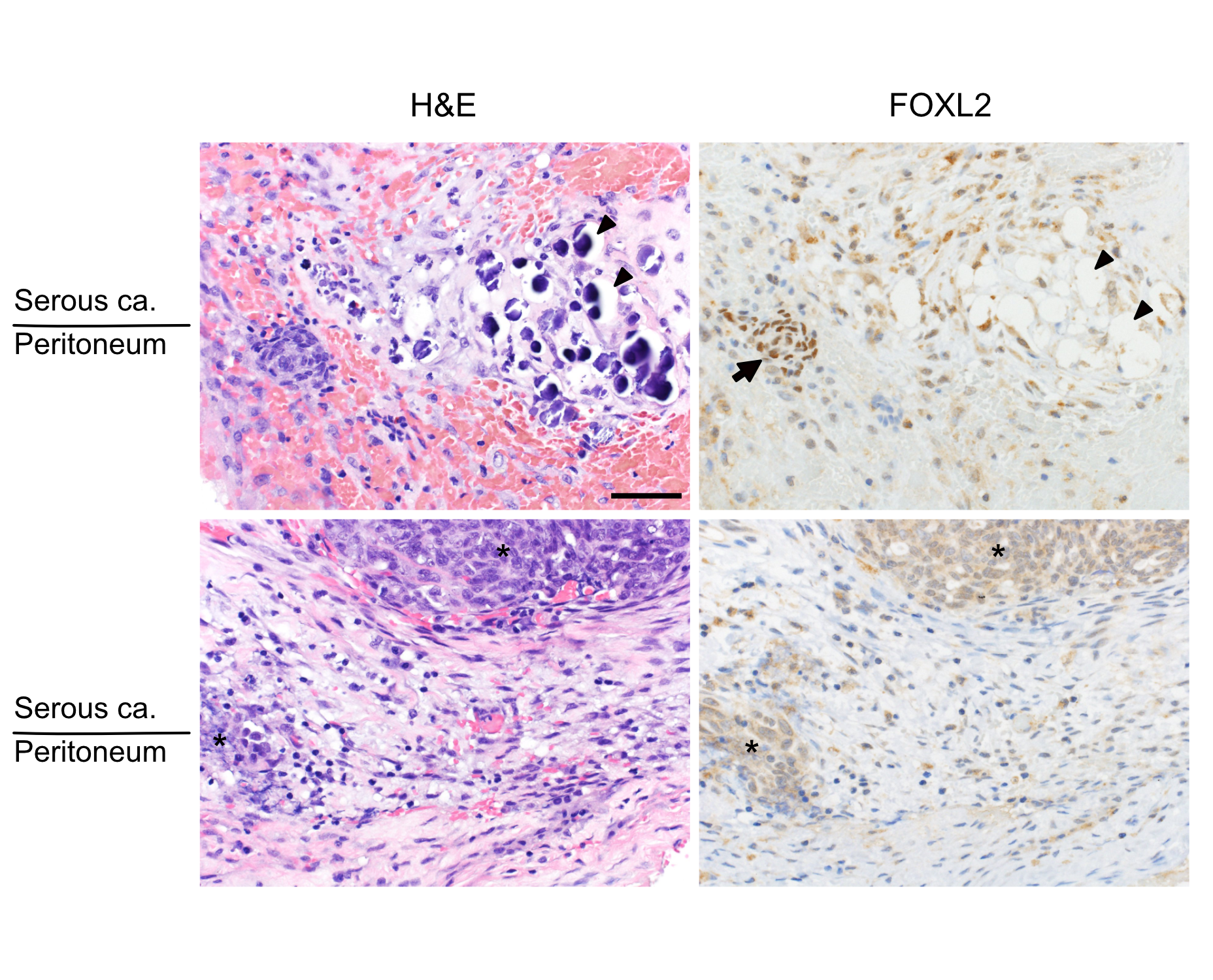

Supplement: S4 Fig — Very few FOXL2-positive cells (arrows) are present near psammoma bodies (arrowheads) whereas most of the stromal cells in this metastatic lesion did not show FOXL2 positivity including those around viable tumor cells (asterisks). The bar indicates 50μm, and the magnification is identical for all the pictures. (TIFF) [file pone.0205494.s004.tiff]
